# Engineering nature for gaseous hydrocarbon production

**DOI:** 10.1186/s12934-020-01470-6

**Published:** 2020-11-13

**Authors:** Mohamed Amer, Helen Toogood, Nigel S. Scrutton

**Affiliations:** EPSRC/BBSRC Future Biomanufacturing Research Hub, Synthetic Biology Research Centre SYNBIOCHEM Manchester Institute of Biotechnology and Department of Chemistry, School of Natural Sciences, BBSRC/EPSRC, The University of Manchester, Manchester, M1 7DN UK

**Keywords:** Synthetic biology, Gaseous hydrocarbons, Propane, Butane, Isobutane, Pathway engineering

## Abstract

The development of sustainable routes to the bio-manufacture of gaseous hydrocarbons will contribute widely to future energy needs. Their realisation would contribute towards minimising over-reliance on fossil fuels, improving air quality, reducing carbon footprints and enhancing overall energy security. Alkane gases (propane, butane and isobutane) are efficient and clean-burning fuels. They are established globally within the transportation industry and are used for domestic heating and cooking, non-greenhouse gas refrigerants and as aerosol propellants. As no natural biosynthetic routes to short chain alkanes have been discovered, de novo pathways have been engineered. These pathways incorporate one of two enzymes, either aldehyde deformylating oxygenase or fatty acid photodecarboxylase, to catalyse the final step that leads to gas formation. These new pathways are derived from established routes of fatty acid biosynthesis, reverse β-oxidation for butanol production, valine biosynthesis and amino acid degradation. Single-step production of alkane gases in vivo is also possible, where one recombinant biocatalyst can catalyse gas formation from exogenously supplied short-chain fatty acid precursors. This review explores current progress in bio-alkane gas production, and highlights the potential for implementation of scalable and sustainable commercial bioproduction hubs.

## Background

One of our biggest global challenges is to reduce the dependence on rapidly diminishing fossil fuels, which impacts climate change and has led to concerns over energy security [1]. This has led to new policies to restrict greenhouse gas emissions, increase the recycling of waste biomaterials and a switch to less polluting renewable alternatives [2]. Biofuels are now well established renewable and sustainable substitutes or additives to conventional transportation and domestic fuels. They are often less polluting, and are derived from biological processes or the chemical conversion of surplus biomass [3]. Bioethanol is the classic example, which is derived from the fermentation of starch or sugars. It is commonly blended with fossil fuels for use in transportation. In contrast, biodiesel is produced chemically via transesterification of plant-derived oils, with a chemical composition similar to conventional diesel [3]. Biologically sourced gaseous biofuels could potentially serve as alternatives to compressed natural gas (CNG) and liquid petroleum gas (LPG). These range from traditional anaerobic digestion (AD)-derived hydrogen and methane [4], to more recently developed de novo pathways to bio-alkane gases (bio-LPG) [5–10].

Successful implementation of commercial biofuel production requires strategies to reduce both polluting waste and the overall carbon footprint from production to usage. Early biofuel developments faced many challenges including the debate over food-fuel competition, the use of clean water resources and the high initial capital and operating costs for large-scale production [11]. Current advanced biofuel strategies attempt to address these challenges by being more economically efficient in terms of net energy gains [12], and are more environmentally sustainable [13]. In addition, advanced biofuels produced by microorganisms have similar properties to petroleum-based fuels. This enables them to ‘drop in’ to existing transportation infrastructures [14], and removes the need for engine modification or the redesign of fuel distribution infrastructures.

Biological solutions to fuels production are often considered to be commercially unviable due to competition with lower cost crude oil and competing synthetic chemistry technologies. In spite of this, in recent years a variety of start-up companies have been established (Table 1) with novel technologies that seek to tackle the issues of cost effectiveness, greenhouse gas mitigation and process efficiency for the production of biofuels [15]. These companies often take advantage of synthetic biology technologies to engineer customised microbial cell factories [16], to enable the de novo production of biofuels from renewable feedstocks. Metabolic engineering tools are employed to modify existing biological pathways and a host microbial ‘chassis’ to maximise productivity.

**Table 1:** Selected benchmark companies making next-generation biofuels

Company/Start up	Technology/description	Website
Photosynthetic		
Algenol	Algal production of ethanol, gasoline, jet and diesel fuels	https://www.algenol.com/
Joule Biotechnologies	Algal production of hydrocarbon-based fuel	www.joulebio.com/
Sapphire energy	Crude oil production from algae	https://sapphireenergy.co.uk/
Synthetic genomics	Conversion of cellulosic biomass into advanced biofuels	https://syntheticgenomics.com/
TerraVia (Solazyme)	Oil production using engineered microalgae from plant-based sugars	https://www.solazyme.com/
Fermentative		
Amyris biotechnologies	Renewable fuels with 80% reduction in greenhouse gas emissions	https://amyris.com/
Gevo	Conversion of cellulosic feedstocks into alternative fuels such as isobutanol	https://gevo.com/
C3 Biotech	Engineering Nature to bio-manufacture hydrocarbon fuels and ethanol from major industrial wastes	https://c3biotech.com/
Global bioenergies	Conversion of waste biomass to glucose for the fermentative production of isobutene	https://www.global-bioenergies.com/
LS9, Inc^a^	Production of biomass-based diesel, renewable chemicals and advanced biofuels	https://www.regi.com
Mascoma	Single step conversion of cellulosic feedstocks into bioethanol using engineered yeast and bacteria	https://www.mascoma.com/
Cell-free enzymatic bioprocess		
Codexis	Conversion of renewable non-food biomass resources into transportation fuels using customised enzymes	https://www.codexis.com/
Synthetic chemistry from non-fossil fuel biomass		
Butamax® advanced biofuels	Biomass conversion into bio-isobutanol production	https://www.butamax.com/
Nesté	Renewable diesel and aviation fuel	https://www.neste.com/en

^a^Life Sustain 9-Billion

One of the most promising new developments is in the design of novel routes to gaseous bio-alkane production (propane and/or (iso)butane), also known as ‘bio-LPG’. This includes the development of chemo-biosynthetic [17] and fully biological (microbial) de novo technologies [5–10]. The focus of this review will be to explore current developments and the potential of biological propane (C3), butane (*n*-C4) and isobutane (*i*-C4) production. This will illustrate how advances in synthetic biology techniques can be employed to modulate native pathways for the accumulation of existing metabolites, and the incorporation of novel pathways to non-native secondary chemicals. Future application of existing technologies could ultimately be extended to longer chain alkanes, thereby tapping into the biodiesel and commodity chemicals markets.

## Gaseous bio-alkanes as biofuels

The utilisation of the liquid biofuels ethanol and biodiesel as transportation fuels is well established globally [14]. Bioethanol is often used as a lead substitute in petrol (gasoline), and is available in blends up to 85% [18]. However, it has only 70% of the energy content of petrol; its purification is energy intensive and its hygroscopic nature leads to corrosion in engines and pipes [19]. Biodiesel has 91% of the energy content of Gasoil (D2 diesel), but it is prone to wax deposition at lower temperatures and current distribution infrastructures are problematic. In contrast, petrol-range alkanes (C3–C9) are promising alternative candidates as biofuels as they have a relatively higher energy density and existing low cost infrastructure is available for liquified gas storage and transportation [20].

Gaseous biofuels production has the advantage over longer chain alkane production as these fuels can be continuously harvested from aqueous microbial cultures due to their hydrophobicity and volatility [6, 14]. The latter is important as liquid biofuels are often cytotoxic to microorganisms, which limits the growth of the host chassis and subsequent biofuels titres. Gas phase harvesting significantly reduces the requirements for costly purification strategies, and downstream liquefaction can occur with existing infrastructure at low pressures.

Biologically-derived methane and hydrogen gases (biogas or ‘coal gas’) are traditionally generated by anaerobic digestion (AD) of municipal, commercial and industrial wastes or energy crops [4]. Biogas is often utilised for the production of both electricity and heat, however a blend of hydrogen and methane can be used in transportation as it reduces exhaust emissions compared with methane alone [21]. Hydrogen is also used in industry for refining, treating metals, and processing food. Biogas streams often require calorific enrichment by the addition of propane, so are less ideal as transport fuels than petrol-range alkanes. In addition, the simplest hydrocarbon gas methane is difficult to liquify and transport [22]. It is also a 20–30 times more potent greenhouse gas than carbon dioxide, and there are worldwide regulations in place to limit the release of emissions that contribute towards global warming. In spite of this, biomethane is predicted to capture a large market share as an alternative transport fuel (~ 14%) and alternative energy fuel (~ 32%) by 2030 [23].

In contrast, propane gas is a highly efficient, clean-burning fuel [2], with existing storage and transportation infrastructure and well-established global markets. It is the third most widely used transportation fuel globally [20 million tons per annum] [6, 24], and is also used as a feedstock for many petrochemical industries [25, 26], domestic heating and cooking, non-greenhouse gas refrigerants and aerosol propellants (C3, *n*-C4 and *i*-C4 blends) [27, 28]. It is currently obtained primarily from natural gas and petroleum refining, and its ‘drop-in’ nature can boost the calorific value of current methane and/or biogas supplies. The only alternative commercial production of propane is the semi-biological Nesté process (Table 1) [17]. This involves a synthetic catalytic conversion of biodiesel waste (glycerol), a by-product of the transesterification of vegetable oils [13]. This energy intensive process is reliant on natural gas derived hydrogen [29], so is not truly a sustainable or renewable process. Therefore, there is a need for the commercial development of sustainable and renewable biological routes to clean burning fuels to allow countries to align with the global strategy of reducing carbon footprint, greenhouse gases and other pollutants.

## Enzymatic alkane production: Deformylation vs decarboxylation

**Fig. 1:**
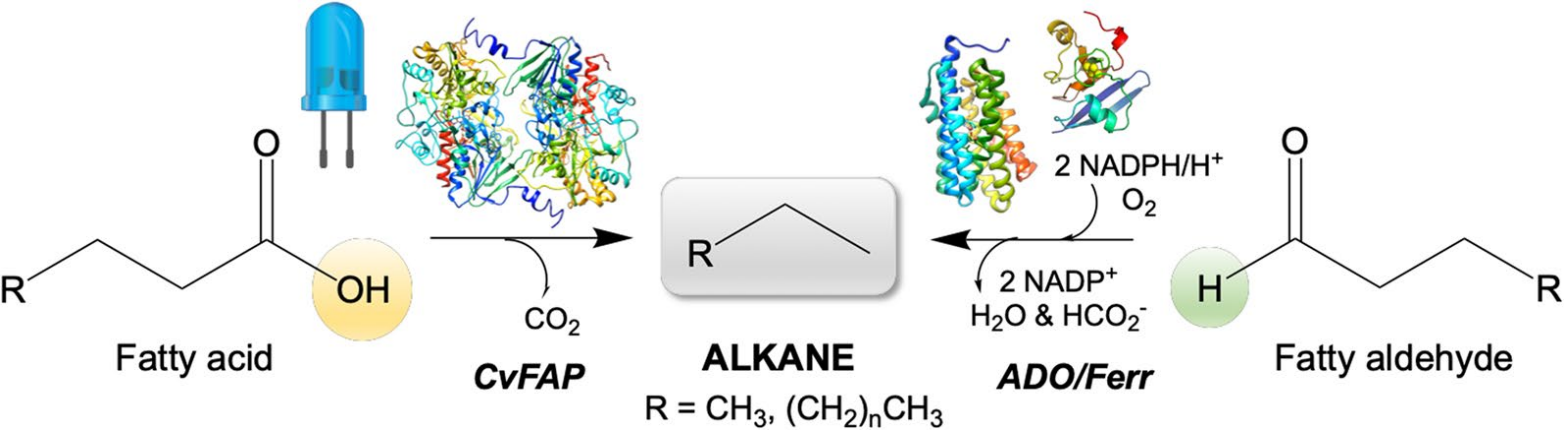
Enzymatic routes to hydrocarbon production using fatty acid photodecarboxylase (CvFAP) and aldehyde deformylating oxygenase (ADO). The crystal structure representations of CvFAP (PDB: 5ncc [35]), ADO (PDB: 4kvr [32]) and ferredoxin (PDB: 1a70 [78]) were generated in Chimera [79]

Biological production of C3, *n*-C4 and *i*-C4 has been observed in trace quantities as secondary metabolites in some microorganisms [7, 28, 30, 31]. However, the natural pathway(s) to short chain alkanes has not been elucidated, and they may instead be by-products of existing pathways present for long chain hydrocarbon biosynthesis. In recent years, two novel enzymes have been exploited for their bio-alkane gas production potential, due to their ability to generate long chain alkanes from fatty acids or aldehydes [6, 8]. The first is aldehyde deformylating oxygenase (ADO), which eliminates formate from fatty C_(n)_ aldehydes to generate the corresponding C_(n-1)_ alkane or alkene (Fig. 1) [32, 33]. The second enzyme is a fatty acid photodecarboxylase (CvFAP), which catalyses the light-dependent decarboxylation of C_(n)_ fatty acids to the corresponding C_(n-1)_ alkane (Fig. 1) [5, 6, 34–36].

ADO and CvFAP are mechanistically and structurally distinct, yet both enzymes display a higher specificity towards medium to long chain fatty aldehydes and acids, respectively [32, 35]. Typical hydrocarbon products of both enzymes are of chain lengths more suitable as petrol or diesel additives [20, 24, 37]. To improve gaseous hydrocarbon production, structurally-driven site-directed engineering studies of both enzymes have been performed to alter their specificity towards shorter chain substrates [6, 32, 38]. This has allowed biocatalytic pathways to be designed for gaseous hydrocarbon production utilising either ADO or CvFAP variants as the terminal enzymatic step. However, future design of commercial enzyme-driven gaseous hydrocarbon production requires an understanding of the catalytic requirements, robustness and efficiency of ADO and CvFAP before deciding whether a terminal deformylation or decarboxylation step is employed.

### Deformylation: aldehyde deformylating oxygenase

The cyanobacterial enzyme responsible for alkane production from fatty aldehydes was initially described as an aldehyde decarbonylase. This was because alkane formation was thought to proceed without any net change in oxidation state [39], with carbon monoxide as the expected co-product [40]. However, sequence and structural analysis of ADO from the cyanobacterium *Prochlorococcus* *marinus* MIT9313 [40] showed it is actually a member of the non-heme dinuclear iron oxygenase family [39], members of which are known to catalyse challenging oxidation reactions. In addition, ADO was shown to require both molecular oxygen and an NADPH-dependent ferredoxin / reductase system for activity to reduce the di-ferric cofactor of ADO to the active di-ferrous state [39, 41]. Later demonstration of formate as the secondary co-product led to the renaming of ADO as an aldehyde deformylating oxygenase (Fig. 1) [41, 42].

The native *P.* *marinus* ferredoxin partner protein for ADO is unknown, however the cyanobacterial homologue PetF from *Synechocystis* *sp.* PCC6803 was found to be a functional alternative [8, 9]. Further studies with an ADO homologue from S*ynechocystis* *elongatus* PCC7942 showed that a fusion with its native ferredoxin: ferredoxin reductase (Fd:FNR) electron transfer complex or the reductase domain of P450RhF from a *Rhodococcus* species successfully generated a self-sufficient enzyme [43].

To increase the specificity of ADO towards gaseous hydrocarbon production, mutagenesis was performed, based on the known crystal structure of ADO from *P.* *marinus* (Joint Center for Structural Genomics; PDB ID: 2OC5) [32, 38]. The substrate binding region of ADO is a tunnel-like pocket lined with hydrophobic residues. The presence of an unknown ligand of extended length in the crystal structure enabled the identification of residues that could potentially influence substrate binding. To increase the binding of butyraldehyde, residues V41 and A134 were targeted by mutagenesis and exchanged for tyrosine and phenylalanine (Table 2) [32]. This strategy was designed to introduce a steric block to longer chain aldehydes beyond a chain length of C9. The most successful variant was A134F, which showed a modest two-fold improvement in the rate of gas production in *E.* *coli* (0.46 mg propane/L culture) compared to wild-type ADO [32]. In another study, multiple substrate channel residues were targeted to introduce steric blocks at strategic places to progressively change the substrate chain length specificity (Table 2). Short chain selectivity improvements were seen with variants A118F and A121F, with increased preference for butyraldehyde and C_4,6,7_ aldehydes, respectively [38].

**Table 2:** Relative In vitro and In vivo studies of ADO and CvFAP for the production of alkane gases

Alkane production (relative *k*_app_ min^−1^)			
Enzyme variant	Propane	Butane	Refs.
ADO kinetic studies relative to wild-type^a^			
A134F	4.5	5.9	[32]
V41Y	1.0	1.8	[32]
A134F/V41Y	1.0	1.3	[32]
A118L	2.2	nd	[38]
A118F	2.2	nd	[38]
A121F	3.3	nd	[38]

Substrate	Decoy	Alkane	Alkane (μM)	Refs.
CvFAP wild-type kinetic studies^b^				
Formic acid	None/C15	H_2_	48.8/291.7	[36]
Acetic acid	None/C14	methane	2.7/7.5	[36]
Propionic acid	None/C13	ethane	103/347.1	[36]
Butyric acid	None/C12	propane	382.2/1090.5	[36]
Isobutyric acid	None/C13	propane	305.2/1007	[36]
Valeric acid	None/C9	butane	860.7/2440	[36]
Isovaleric acid	None/C9	isobutane	268.6/940.2	[36]

Alkane production (mg/L)					
Enzyme variant	Propane		Butane	Isobutane	Refs.
ADO In vivo production of alkane gases^c^					
Wild-type	0.27		nd	nd	[32]
A134F	0.46		nd	nd	[32]
CvFAP In vivo production of alkane gases in E. coli^d^					
Wild-type	7.0		17.7	5.6	[6]
G462A	17.6		33.5	30.2	[6]
G462F	31.2		27.7	28.6	[6]
G462I	43.8		47.1	86.8	[6]
G462V	24.5		21.9	17.4	[6]
CvFAP In vivo production of alkane gases in other microorganisms^e^					
G462I in *Halomonas* TQ10		7.0	17.7	5.6	[6]
G462I in *Synechocystis*		17.6	33.5	30.2	[6]

*nd* not determined

^a^Kinetic studies of isolated enzymes in the presence of butyraldehyde or valeraldehyde using a chemical reducing system (ferrous ammonium sulphate, phenazine methosulphate and NADH). Data is expressed as relative *k*_app_ (min^−1^) compared to wild-type enzyme

^b^Kinetic studies of CvFAP isolated enzymes with 150 mM substrate ± a decoy molecule

^c^In vivo production of propane with ADO co-expressed with ferredoxin in *E.* *coli*. Cultures were grown in the presence of 10 mM butyraldehyde

^d^*E.* *coli* cultures expressing CvFAP in the presence of supplemental 10 mM butyric, valeric or isovaleric acid

^e^Cultures expressing CvFAP_G462I_ in the presence of 80 mM butyrate or CO_2_ for *Halomonas* or *Synechocystis*, respectively

The biotechnological application of ADO in scaled bio-alkane production is hampered by the relatively poor efficiency of the enzyme (turnover number of ~ 3–5 h^−1^) [32], even with the preferred long chain fatty aldehydes [8, 9, 20]. For example, under steady state turnover conditions the reaction with heptanal has a *k*_cat_ of ~ 1 min^−1^, in spite of the report of an exponential burst phase with a *k*_app_ of 0.27 s^−1^ [32, 42]. In addition, this enzyme requires the co-expression of ferredoxin to supply an electron transfer system. For bio-alkane production, upregulation and subsequent accumulation of fatty aldehyde precursors is problematic due to the reactive and toxic nature of these compounds [9, 20, 44, 45]. In spite of this, multiple studies have demonstrated successful moderate production of gaseous and non-gaseous hydrocarbons in *Escherichia* *coli* and other microorganisms [5, 8–10, 20, 46].

### Decarboxylation: fatty acid photodecarboxylase

The discovery of a fatty acid photodecarboxylase from the algae *Chlorella* *variabilis* NC64A (CvFAP) enabled the development of secondary ADO-independent biological routes to hydrocarbon production [35]. This blue light-activated, FAD-containing enzyme is a member of the glucose–methanol–choline (GMC) oxidoreductase family of enzymes. The mechanism of action of CvFAP is currently under investigation, but structural and spectroscopic studies suggest it likely proceeds via a radical-based decarboxylation of fatty acids, with the release of carbon dioxide as the co-product (Fig. 1) [34–36, 47, 48]. Unlike ADO, this enzyme does not require a secondary electron transfer partner, nicotinamide cofactor or molecular oxygen for activity. It reputedly has a very promising turnover number of up to 8000 [35], suggesting it has a higher potential than ADO for scaled hydrocarbon production [6].

CvFAP shows a marked preference for long-chain fatty acids (C12−C20), with demonstrated substrate/product conversion of 96% for both C16 and C17 acids [34]. Activity drops dramatically for medium chain (C12) carboxylic acids (11% conversion), with only poor activity seen with C3-C4 acids [6, 36]. One approach taken to increase wild-type CvFAP activity towards shorter chain substrates was to introduce a decoy molecule to ‘fill up’ the vacant space in the substrate access channel [36]. It was found that the decoy molecule did not facilitate the binding of the carboxylic acid, but rather increased the enzyme reaction rate. This successfully led to an increase in activity with medium and short chain substrates (Table 2). For example, propane production from butyric or isobutyric acid increased nearly three-fold when reactions were supplied with C12 or C13 alkanes as decoy molecules, respectively [36].

The crystal structure of CvFAP revealed that like ADO, it contained a hydrophobic substrate access channel that was designed to accommodate long chain fatty substrates [35]. Mutagenesis was performed to add a steric block to the substrate access channel to increase the specificity towards C3-C4 acids [6]. An important discriminating residue was found to be G462, as substitution to valine and isoleucine increased propane production 5- to 16-fold from exogenously supplied butyric acid (3.4 and 10.8 mg propane per L culture, respectively; Table 2) [6].

The simpler reaction requirements and the higher turnover number of CvFAP compared to ADO suggest the former is a better candidate for scaled hydrocarbon gas production. However, a major limitation of CvFAP is its inherent instability, as seen by a loss of flavin content during protein purification [47]. Studies revealed that blue light-exposure, which is necessary for CvFAP activity, also irreversibly inactivates the enzyme through the formation of protein based organic radicals [47]. Photoinactivation was especially evident in the absence of a bound fatty acid substrate. In addition, continuous blue light exposure is known to be cytotoxic to microorganisms [49], therefore any CvFAP-dependent continuous culture strategies would need periodic ‘dark phases’ to enable culture (and enzyme) replenishment [6]. However, while neither ADO nor CvFAP are ideal biocatalysts, studies have shown that both can potentially be used as terminal biocatalysts in microbial hydrocarbon gas production strategies.

## Engineered biological routes to alkane gases

**Fig. 2:**
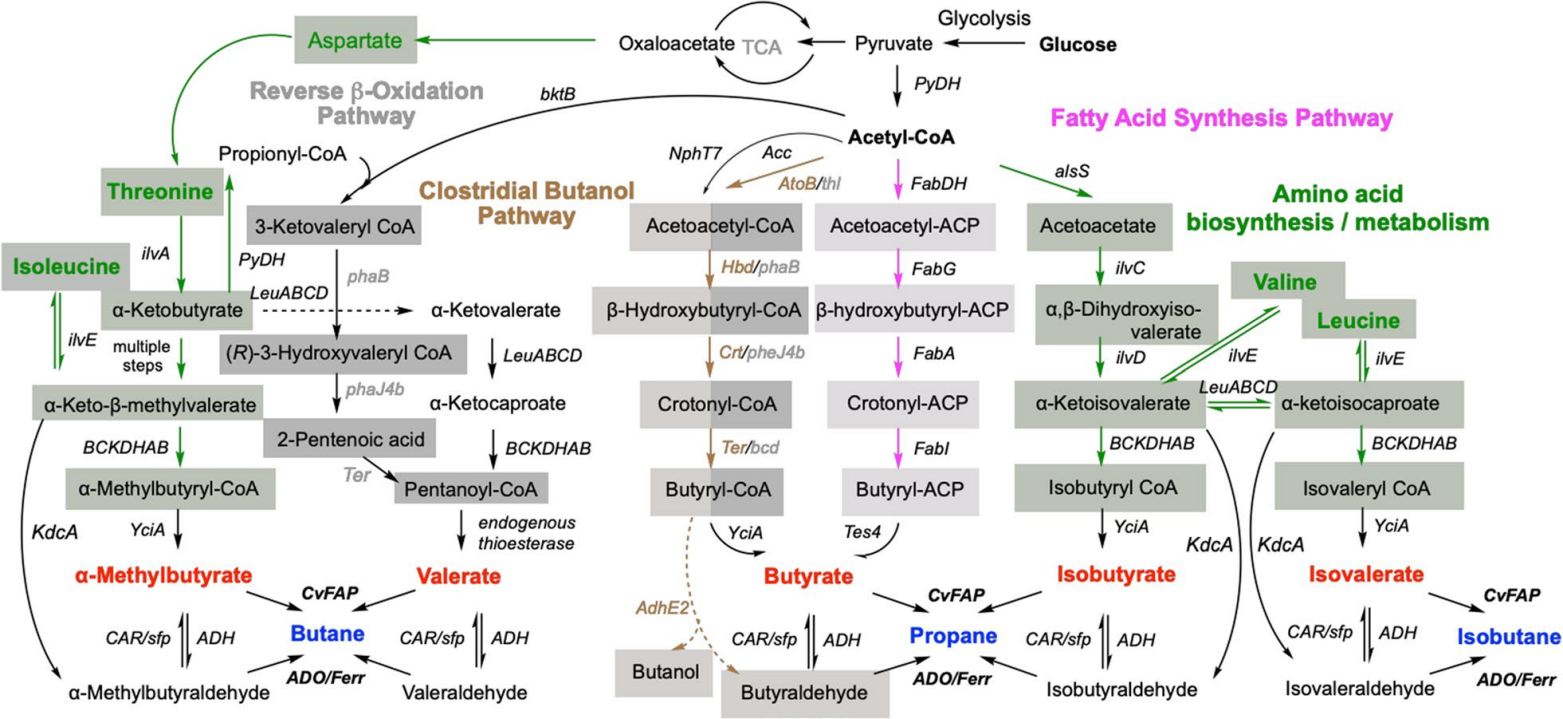
Biosynthetic pathways developed for the production of propane, butane and isobutane. Chemicals highlighted in red and green are bio-alkane gas precursors that could be fed into cultures for single step CvFAP-dependent and amino acid metabolism pathways, respectively. Enzymes: *Acc*: acetyl-CoA carboxylase; ADH: Aldehyde dehydrogenase homologue; *AdhE2*: bi-functional aldehyde-alcohol dehydrogenases; *ADO/Ferr*: aldehyde-deformylating oxygenase and electron transfer partner ferredoxin; *alsS*: acetolactate synthase; *AtoB*: acetyl-CoA acetyltransferase; *bcd*: butyryl-CoA dehydrogenase; BktB: β-ketothiolase; *BCKDHAB*: human 2-oxoisovalerate dehydrogenase; *CAR/sfp*: carboxylic acid reductase and activating enzyme 4′-phosphopantetheinyl transferase; *Crt*: 3-hydroxybutyryl-CoA dehydratase; *CvFAP*: fatty acid photodecarboxylase; *FabA*: β-hydroxyacyl-ACP dehydrase; *FabDH*: malonyl-CoA-ACP transacetylase and β-ketoacyl-ACP synthase; *FabG*: β-ketoacyl-ACP reductase; *FabI*: enoyl-ACP reductase; *Hbd*: 3-hydroxybutyryl-CoA dehydrogenase; *ilvA*: L-threonine dehydratase biosynthetic; *ilvC*: ketol-acid reductoisomerase; *ilvD*: dihydroxy-acid dehydratase; *ilvE*: branched-chain amino acid aminotransferase; *KdcA*: branched-chain keto acid decarboxylase; *LeuABCD*: isopropyl malate synthase, dehydrogenase & isomerases complex; *NphT7*: acetoacetyl CoA synthase; *phaB*: acetoacetyl-CoA reductase; *phaJ4b*: R-specific enoyl CoA hydratase 4b; *PyDH*: pyruvate dehydrogenase; *Ter*: trans-2-enoyl-CoA reductase; *Tes4*: acyl-ACP thioesterase; *thl*: thiolase; *YciA*: acyl-CoA thioester hydrolase

Multiple de novo routes to bio-alkane gas production have been developed, the majority utilising a terminal ADO-dependent deformylation of fatty aldehydes (Fig. 2). However, the discovery of CvFAP has opened up new routes to alkane gas production, some of which are potentially truncated versions of ADO-dependent pathways. These routes are based on a single step process [6, 32, 36], fatty acid biosynthesis [8], clostridial butanol pathway / reverse β-oxidation [9, 20], valine biosynthesis [10] and amino acid catabolism pathways [5, 6] (Fig. 2).

With the exception of single step biocatalytic strategies, In vivo bio-alkane pathway design is based on the upregulation or introduction of non-native routes to fatty aldehyde or acid precursors. These fully biological routes have been investigated primarily within *E.* *coli*, but the potential usefulness of other microbial hosts have also been explored [6]. The following sections will describe current and potentially new strategies for bio-alkane gas or bio-LPG production.

### Single step bioprocesses

Single step microbial bioprocesses can be advantageous over cascading pathways or In vitro reactions as only one biocatalyst is needed, and coenzymes and accessory proteins can be produced or recycled in situ where needed. However, any commercially successful scaled bio-alkane gas process would require high precursor loading, so the cytotoxicity of the fatty aldehyde or acid substrate would need to be mitigated against.

Recently, sustainable and renewable solutions to single step bio-alkane production (tuneable bio-LPG) were demonstrated using CvFAP variants expressed in robust microbial hosts [6]. In this study, a combinatorial approach was taken whereby proof of principle demonstration was followed by microbial chassis screening and development, design of scaled bio-LPG production ‘hubs’, and techno-economic analysis of the bioprocess to improve the commercialisation potential. The latter included sourcing local waste materials for both carbon and fatty acid supply, such as AD of crop residue and household food waste. This approach could positively impact on global carbon management targets and clean air directives by the valorisation of both industrial and domestic waste [6].

The proposed bio-LPG hub design was based on supplementing microbial cultures with specific mixtures of butyric and valeric acids, to generate customer-specific blends of propane and butane as an LPG substitute. A variety of CvFAP variants were screened in *E.* *coli*, the most successful of which was CvFAP_G462I_. Cultures grown in the presence of supplemented butyrate, valerate and isovalerate generated the highest titres of propane, butane and isobutane (43.8, 47.1 and 86.8 mg/L), respectively (Table 2). Transitioning of CvFAP_G462I_ into the robust halophilic industrial bacterium *Halomonas* should enable a more commercially viable process to be developed. Cultures were grown in batch or continuous culture under non-sterile conditions in seawater or wastewater on biodiesel waste as a feedstock [6]. A pilot scale bioprocess hub was proposed, based on a 10 m^3^ dark biomass generator (no light), two 10 m^3^ photobioreactors for gas generation, and a 17 m^3^ AD plant to supply the fatty acids. Cultures would require cyclic residence in the dark and light tanks to maintain cell density and maximise productivity. Gas production in *Halomonas* was found to be lower than in *E.* *coli* (Table 2), however techno-economic analysis of the proposed pilot scale bioprocess hub when scaled up further (by ten-fold) was predicted to generate around 358 tonnes per year [6].

The ultimate ‘carbon neutral’ approach to bio-alkane gas production would be to utilise industrial CO_2_ effluent as the carbon source by transitioning CvFAP into a photosynthetic host. Recently, CvFAP_G462I_ was expressed in the photosynthetic algae *Synechocystis* PCC 6803 [6], which had been chromosomally modified to increase internal butyrate production [50]. In spite of elevation of In vivo butyrate production in this strain [6, 50], propane production was more pronounced when cultures were supplied with external butyrate (17.6 mg/L; Table 2). Therefore, *Synechocystis* cultivation from waste CO_2_ could be utilised as a butyrate supply for *Halomonas*-dependent bio-alkane gas production as an alternative to requiring AD plants to supply fatty acids [6].

There has been only a limited number of studies to explore the potential of single-step ADO-dependent propane production. *E.* *coli* expressing ADO wild-type and the A134F variant were cultivated in the presence of 10 mM butyraldehyde (Table 2) [32]. In this case, propane titres were relatively low (0.27 and 0.46 mg/L, respectively) compared to comparable CvFAP-containing cultures.

### Heterologous butyraldehyde upregulation via the fatty acid biosynthesis (FAB) pathway

Multi-step pathways to secondary metabolite production are designed to increase In vivo precursor biosynthesis by upregulating existing pathways and/or the introduction of non-native biocatalysts. In the case of ADO, the immediate precursor for propane production is butyraldehyde. One route to butyraldehyde is via the incorporation of carboxylic acid reductase (CAR) from *Mycobacterium* *marinum*, activated by maturation factor protein (sfp; *Bacillus* *subtilis*), which catalyses the reduction of butyric acid to butyraldehyde [51]. Therefore, it is not surprising that the first described route to propane production in *E.* *coli* was a modification of the native fatty acid biosynthesis pathway (FAS) [8]. As butyric acid is the direct precursor of CvFAP, ADO-dependent pathways can potentially be modified to eliminate CAR/sfp and substitute ADO/Ferr for a terminal light dependent decarboxylation step (Fig. 2).

**Fig. 3:**
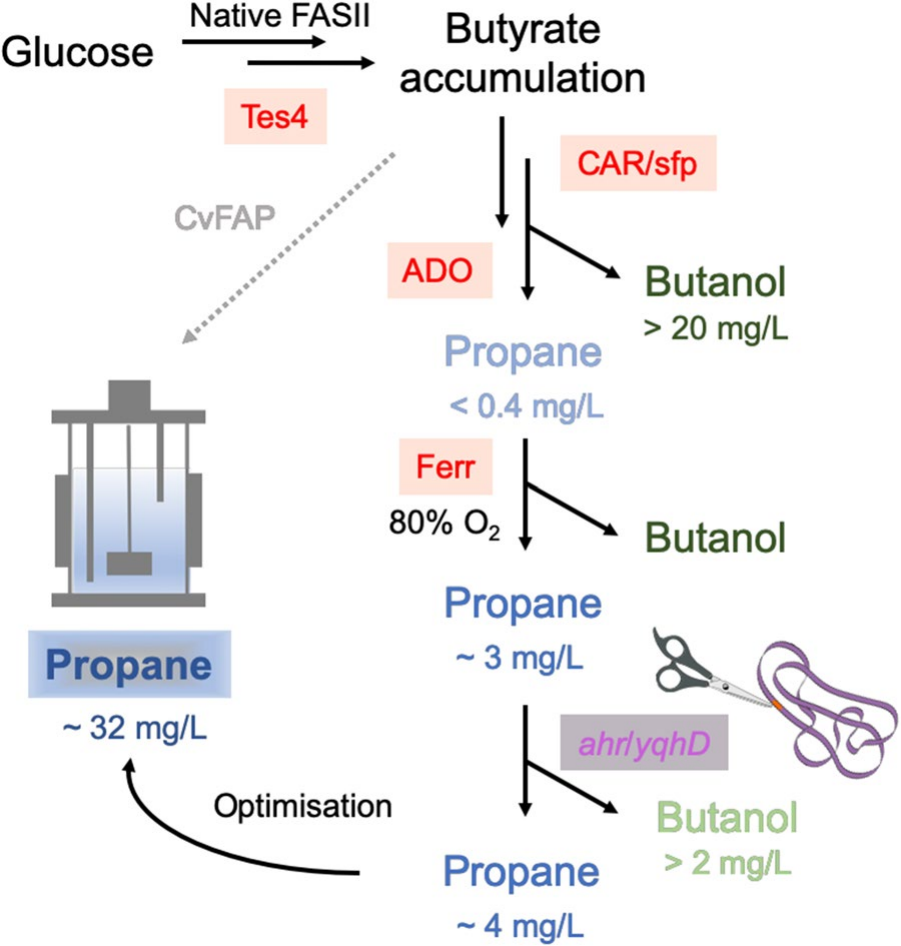
Development of a de novo pathway to propane production in *E.* *coli* based on fatty acid biosynthesis. Non-native genes inserted into *E.* *coli* are highlighted in red. Native aldehyde dehydrogenase genes chromosomally deleted are highlighted in magenta. The grey dotted line indicates a potential shortened pathway strategy that has not been tested. Data were obtained from [8]

The FAS pathway involves a series of acyl carrier protein (ACP)-dependent chain elongation steps beginning with acetyl-CoA (Fig. 2) [52]. The first stage in FAS modification was to bypass fatty chain elongation steps beyond butyryl-ACP by the introduction of thioesterase Tes4 from *Bacteroides* *fragilis* (Fig. 2) [8, 53]. This is based on the cyanobacterial route to alkane biosynthesis, whereby fatty acyl-ACP molecules are converted into fatty acids by acyl-ACP reductases [40]. This successfully led to butyrate accumulation in *E.* *coli* (Fig. 3). The addition of the enzymes CAR, sfp and ADO led to low titres of propane production (~ 0.4 mg/L), with significantly higher levels of the by-product butanol detected (~ 20 mg/L) [8]. The latter is generated via the native aldehyde detoxification mechanism, where endogenous aldehyde reductases convert butyraldehyde into butanol [54]. Propane titres increased around seven-fold by the inclusion of Ferr from *Synechocystis* sp. PCC 6803 to act as an electron transfer partner for ADO. Additional optimisation strategies tested were increasing the culture oxygen concentration and the inclusion of an NADPH / ferredoxin/flavodoxin-oxidoreductase (Fpr); the latter to ensure Ferr reduction was not limiting in *E.* *coli* [55]. Microbial chassis modification was performed by the chromosomal deletion of aldehyde reductases *ahr* and *yqhD*, which led to a ten-fold reduction in by-product butanol formation. Finally, further optimisation of cultivation conditions led to a maximal titre of ~ 32 mg/L propane in *E.* *coli* [8].

Bacteria expressing CvFAP variants are known to generate alkane gases when cultivated in the presence of volatile fatty acids (VFAs) [6]. However, a similar approach with ADO / Ferr could be envisioned by the co-expression of CAR and sfp to convert the VFAs into the corresponding fatty aldehydes. This approach was tested in *E.* *coli* to generate C3-C7 alkanes, using a variety of cyanobacterial ADO orthologues [33]. The best performing ADO in most cases was from *Nostoc* *punctiforme* PCC 73,102, which generated propane in the presence of butyric acid at an initial rate of 20–23 μmol/L/OD_600 nm_/h. Overall propane yields were higher with this orthologue compared to the *P.* *marinus* ADO variant A134F [33]. Other studies have also described the production of alkanes from modifications of the FAS pathway, but they were limited to mostly medium and long chain hydrocarbons (min C5) [20, 56, 57].

### Pathways based on reverse β-oxidation and Clostridial butanol production

**Fig. 4:**
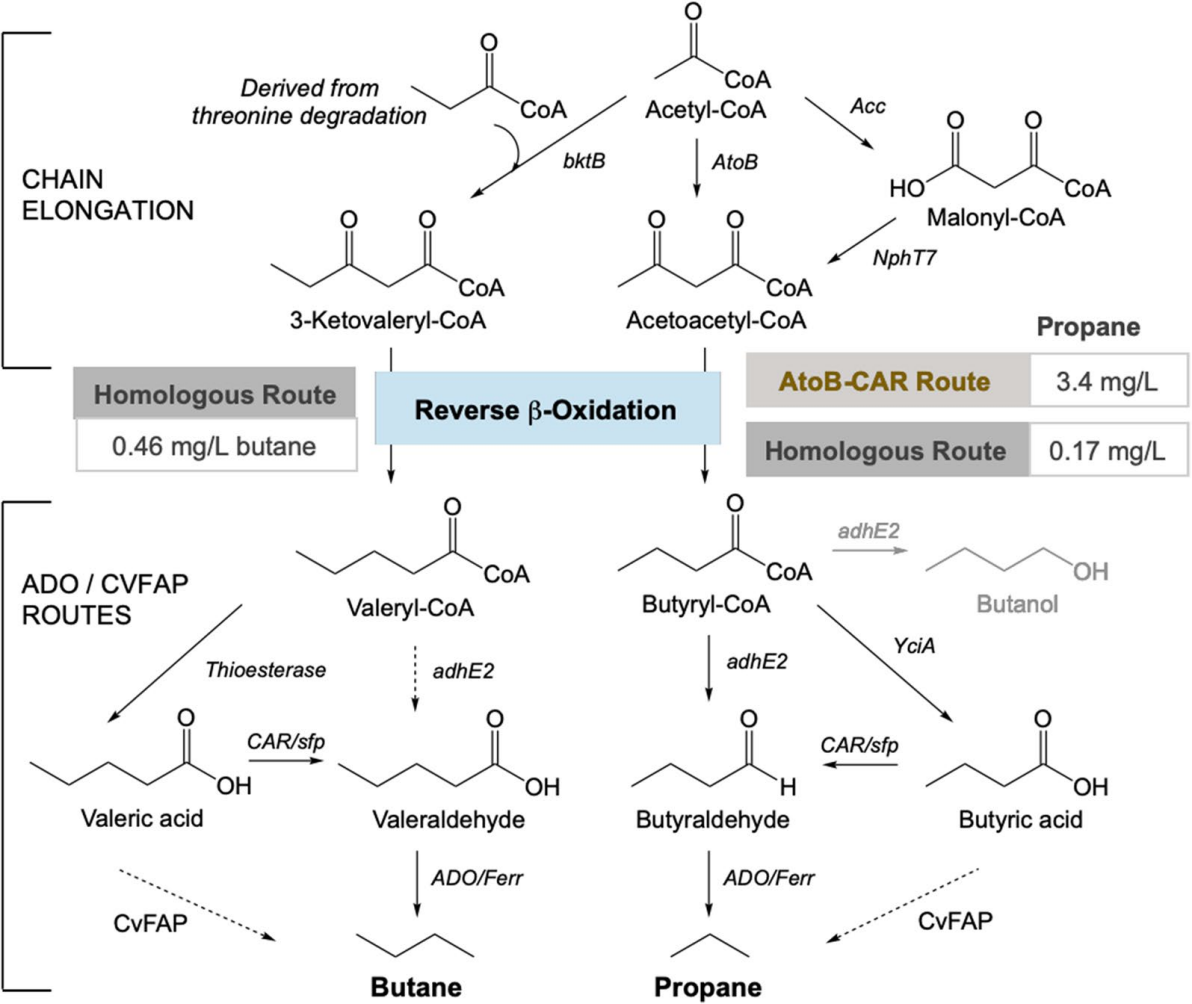
CoA-dependent pathways to propane and butane via clostridial butanol pathway [9] and reverse β-oxidation [20, 59]. The homologous route is constructed with enzymes from *Cupriavidus* *necator* and *Treponema* *denticola*. Dashed arrows indicate proposed alternative routes not currently tested. *adhE2*: = bi-functional aldehyde-alcohol dehydrogenase. Other enzymes are defined in the Fig. 2 legend

A parallel route from acetyl-CoA to butyrate has been engineered based on the CoA-dependent reverse β-oxidation pathway for fatty acid breakdown [58–60]. This pathway is important within solventogenic Clostridia, as it is used for the production of butanol (Fig. 2) [61]. The engineered alkane pathway is initiated by the condensation of two acetyl-CoA molecules to acetoacetyl-CoA. This can be catalysed directly by *E.* *coli* acetyl-CoA acetyltransferase (atoB), or indirectly via hydroxyl removal from malonyl-CoA by *Streptomyces* acetoacetyl-CoA synthase (NphT7; Fig. 4) [9]. This is followed by three consecutive steps to butyryl-CoA catalysed by 3-hydroxybutyryl-CoA dehydrogenase (Hbd), 3-hydroxybutyryl-CoA dehydratase (Crt) and *trans*-2-enoyl-CoA reductase (Ter; Fig. 2). This is analogous to the FAS-route from acetoacetyl-ACP to butyryl-ACP, except that acyl carrier protein is substituted for coenzyme A. Butyrate formation from butyryl-CoA was achieved via the incorporation of the acyl-CoA thioester hydrolase (YciA) from *Haemophilus* *influenza* [9]. The remaining steps to propane are the same as the YciA and ADO_A134F_-dependent FAS route, co-expressing the accessory enzymes sfp and Ferr, respectively. This route also has the potential to be truncated at butyric acid production, allowing propane to be produced by the inclusion of CvFAP (Fig. 2).

The most successful route in *E.* *coli* was found to be the clostridial atoB-CAR version (3.4 mg/L propane) [9]. These titres were achieved using the *E.* *coli* aldehyde dehydrogenase knockout strain (Δ*ahr*/Δ*yqhD*) for reduced butanol by-product formation [8]. An alternative to YciA/CAR-dependent butyraldehyde production is the incorporation of bi-functional aldehyde-alcohol dehydrogenase adhE2 from *C.* *acetobutylicum* [62]. This enzyme directly converts butyryl-CoA into butyraldehyde, however it subsequently catalyses butanol production. Therefore, this route is likely to increase butanol production at the expense of propane titres.

An additional version of this pathway was described in *E.* *coli*, with the genes encoding atoB, Hbd, Crt and Ter substituted for homologues from *Cupriavidus* *necator* and *Treponema* *denticola* (Fig. 2) [20]. However, titres were lower than those utilising the clostridial enzyme route (0.17 mg/L). This may be in part due to the absence of YciA, as this system relied on the presence of an endogenous *E.* *coli* thioesterase [20].

Butane production was also achieved using the reverse β-oxidation route, beginning with the condensation of acetyl-CoA and propionyl-CoA to form 3-ketovaleryl-CoA [20] (Fig. 4). This is catalysed by β-ketothiolase from Cupriavidus necator (BktBCn), and is analogous to the atoB-catalysed condensation of two acetyl-CoA molecules to form acetoacetyl-CoA. The remainder of the pathway to butane follows the same reverse β-oxidation route with CAR and ADO (Fig. 4). Using an endogenous thioesterase (no YciA) and wild-type *E.* *coli*, titres of 0.35 mg/L butane were obtained. This increased to 0.46 mg/L when the RARE *E.* *coli* strain was used, which has undergone genomic modification to delete six native aldehyde reductases [63].

Given that the FAS and reverse β-oxidation routes share a common precursor (acetyl-CoA) and analogous routes to fatty acid precursors, the efficiency and energy burden of each pathway has been examined. Overall, the reverse β-oxidation route is theoretically presumed to be the most efficient route to fatty acids in terms of cofactor balancing and energy consumption [20]. FAS reductases are in general thought to prefer NADPH [64], while the CoA-dependent β-oxidation enzymes are NADH dependent [65]. Increasing NADPH usage through the FAS-based pathway would increase the carbon flux to the pentose phosphate pathway, unless the host strain contains an efficient engineered transhydrogenase. The FAS pathway also requires the utilisation of ATP, reducing the overall energy efficiency of the process. There is also a greater potential in transferability of a reverse β-oxidation pathway into multiple microorganisms, as it is dependent on the universal CoA molecule rather than an organism-specific ACP [66].

### Bio-alkane gas production from de novo amino acid biosynthesis and catabolism pathways

A third set of pathways has been developed for alkane gas production based on selected amino acid biosynthesis and/or catabolic routes. Amino acids were selected based on the composition of their respective *R*-groups. For example, the deamidation and decarboxylation of valine, isoleucine and leucine would essentially generate propane, butane and isobutane, respectively [5].

A modified valine biosynthetic pathway for propane production was designed [10] based on prior studies that generated a non CoA-dependent pathway for isobutanol production in *Saccharomyces* *cerevisiae* [67], *S.* *elongatus* PCC 7942*,* [67] and *B.* *subtilis* [68]. Beginning with acetyl-CoA, the initial three steps to α-ketoisovalerate were upregulated by the inclusion of the recombinant genes encoding *B.* *subtilis* acetolactate synthase (*alsS*) and *E.* *coli* ketoacid reductoisomerase and dihydroxyacid dehydratase (*ilvC* and *D*) genes (Fig. 2). At this point the valine biosynthesis pathway was diverted by the inclusion of an α-keto-acid decarboxylase from *Lactococcus* *lactis* (Fig. 2) [10]. This enzyme catalyses the decarboxylation of α-ketosiovalerate to form isobutyraldehyde [67]. To minimise the production of the side product isobutanol from isobutyraldehyde, the host *E.* *coli* strain was engineered to knock out nine aldehyde reductase genes (strain BW25113(DE3)Δ13), including *YqhD* and *Ahr*. This knock out strain successfully increased in isobutyraldehyde accumulation from 0.3 to 1.1 g/L, while isobutanol levels dropped from 0.57 g/L to very low levels [10]. In the final step, ADO catalysed the deformylation of isobutyraldehyde to generate propane, relying on the presence of an *in-situ* electron donor partner.

To increase propane yields, ADO mutagenesis studies were performed to add a steric block to longer chain substrates (A134 and V41 to F or Y), and broaden the substrate channel to accommodate branched chain substrates (I127, A48, A131, Y135, Q123, F100, I37 and I40 to G or A) [10]. In vivo studies with wild-type *E.* *coli* showed an increase in propane production only in variant I127G (83%) compared to wild-type enzyme. Subsequent studies with the BW25113(DE3)Δ13 strain yielded titres of 267 μg/L propane (Table 3), which is three-fold higher than the wild-type ADO constructs (91 μg/L) [10]. However, the maximal propane titre obtained here are 164-fold lower than the levels obtained in *E.* *coli* expressing CvFAP_G462I_ in the presence of supplemental butyric acid (Table 2).

**Table 3:** Microbial alkane gas production via amino acid biosynthesis and/or degradation routes

Alkane production (mg/L)				
Terminal Enzyme	Propane	Butane	Isobutane	Comments
KdcA-dependent pathway in E. coli (valine biosynthesis) [10]				
ADO_WT_	0.091	na	na	*E.* *coli* BW25113(DE3)Δ13 strain
ADO_I127G_	0.267	na	na	*E.* *coli* BW25113(DE3)Δ13 strain
KdcA-dependent pathway in E. coli (amino acid catabolism) [5, 6]				
CvFAP_G462I_	7.8	7.6	48.7	Endogenous amino acids
CvFAP_G462I_	109.7	142	112.1	30 mM amino acids supplied
KdcA-dependent pathway in Halomonas (amino acid catabolism) with added amino acids [5, 6]				
CvFAP_G462I_	8.0	0.11	0.65	Plasmid-borne construct
CvFAP_G462I_	2.7	0.04	0.29	Genomic integrated construct
CoA-dependent pathways in E. coli (amino acid catabolism) [5]				
ADO_A134F_	0.96	0.03	0.01	Endogenous amino acids
CvFAP_G462I_	0.98	0.09	0.04	Endogenous amino acids

In vivo studies determined in the presence or absence of supplemental amino acids (30 mM)

*na* not applicable

An alternative approach is to supplement cultures with specific amino acids, and engineer the microorganism with the degradative pathway to the respective fatty aldehyde (or acid) precursor. This approach was utilised for the production of propane, butane and isobutane by recombinant *E.* *coli* in medium supplementated with valine, isoleucine and leucine, respectively [5, 6]. The commercial potential of this approach is great, as the abundance of protein-rich wastes ensures a cost-effective amino acid and carbon source supply for microorganism cultivation. By adjusting the relative concentrations of each amino acid in the culture medium, tuneable customer-specific alkane gas blends could potentially be generated.

The amino acid degradation approach requires the same set of four biocatalytic enzymes to generate all three alkane gases In vivo, with the relative alkane proportion dependent on the concentrations of each individual amino acid present in the culture medium. The degradation pathway was initiated by amino acid deamination catalysed by leucine 2-oxoglutarate transaminase (ilvE) from *E.* *coli* [69] to form the respective α-keto fatty acid (e.g. α-ketoisovalerate from valine; Fig. 2). This is followed by KdcA-dependent decarboxylation to the respective fatty aldehyde. In this study, the terminal step for alkane gas production was a CvFAP-dependent decarboxylation of fatty acids (isobutyric acid for propane) rather than ADO-catalysed deformylation of the fatty aldehyde. This required an additional step, where the fatty aldehyde was converted to the respective fatty acid by 3-hydroxypropionaldehyde dehydrogenase (Hpad) from *E.* *coli* [70].

The KdcA-dependent amino acid degradation pathway to alkane gas production was initially tested in the *E.* *coli* Δ*ahr*/Δ*yqhD* knockout strain with CvFAP variants G462I and G462V [5, 6]. Both generated similar levels of each alkane gas when amino acids were supplied to the culture medium, however there was a slight increase with the G462I variant. For example, titres of propane, butane and isobutane were 109.7, 142 and 112.1 mg/L in the presence of 30 mg/L valine, isoleucine and leucine, respectively. These levels are more than 400-fold improved over the best ADO_I127G_-dependent valine biosynthesis-based route to propane production (Table 3). In the absence of amino acid supplementation, the alkane gas titres drop to around 8 mg/L for propane and butane, with a noticeable preference for isoleucine production (48.7 mg/L) [5, 6]. The dramatic differences in alkane gas titres between valine biosynthetic and amino acid degradation routes is likely due to multiple factors, such as the substitution of ADO for CvFAP and dramatic increases in supplemental precursor supply in the latter case.

The commercial potential of the CvFAP_G462I_-dependent amino acid degradation pathway was explored by transitioning the four-gene construct into *Halomonas* [5, 6]. Alkane gas production dropped significantly (8 mg/L propane; Table 3), potentially due to differences in the expression of active recombinant enzymes between the two organisms, dramatic differences in culture conditions and amino acid uptake rates. A single-site genome-integrated construct was tested, yielding even lower titres of propane (2.7 mg/L). However, lab-scale non-sterile fermentation studies of *Halomonas* (400 mL) supplemented with 1.8% valine generated 89 mg propane/g cells, mostly within the first 24 h [5]. The longevity of the bioprocess was increased by using an inducible genome-integrated construct, with propane titres increasing gradually to a peak at 70 h (*ca* 180 mg/g cells/day), followed by a slow decline to minimal titres by around 140 h. Switching from an IPTG-inducible to a constitutive system led to fairly consistent propane production rates of around 30 mg/g cells/day for up to 8 days [5, 6].

A second CoA-dependent amino acid degradation pathway has been designed, which contains the same initial ilvE deamination step as the KdcA-dependent route [5]. This pathway then diverges by the introduction of the human branched-chain α-keto acid dehydrogenase complex (BCKDHAB) [71], which catalyses a CoA-dependent decarboxylation of α-keto fatty acids in two-successive steps to form the corresponding acyl-CoA (Fig. 2). For example, in the presence of valine, enzymes ilvE and BCKDHAB results in isobutyryl-CoA production. This is similar to butyryl-CoA generated using the β-oxidation pathway, but distinct from isobutyraldehyde generated by KdcA via non-CoA dependent decarboxylation. The acyl-CoA precursors then undergo the same YciA/CvFAP or YciA/CAR/sfp/ADO/Ferr alternative routes to generate alkanes, as described for the β-oxidation pathway [5].

In vivo *a*lkane gas production by the CoA-dependent amino acid degradation routes were tested in *E.* *coli* strain Δ*ahr*/Δ*yqhD* in the absence of amino acid supplementation [5]. Alkane gas titres from both ADO- or CvFAP-dependent pathways were significantly lower than those obtained for the KdcA-dependent pathways with CvFAP cultivated under similar conditions (< 1 mg/L propane; Table 3). In this case, the major gas produced was propane instead of isobutane [5].

Theoretical pathways from threonine to butane have been proposed, which differ from the KdcA- and CoA-dependent pathways [5, 20]. This is because threonine is a polar, uncharged amino acid with a side chain containing a hydroxyl group rather than a hydrocarbon chain. Therefore, the utilisation of threonine to form butane would require carbon chain elongation steps and hydroxyl removal in addition to deamination and decarboxylation. Threonine has already been implicated in the β-oxidation pathway from acetyl-CoA to propane (Fig. 2 and 4). This is via degradation of threonine to propionyl-CoA, which is a co-substrate of bktB with acetyl-CoA in the first step to form 3-ketovaleryl-CoA [20].

The first proposed route is based on upregulating the native threonine to isoleucine biosynthetic pathway [72], except for the terminal transamination step, to allow an accumulation of α-keto-β-methylvalerate. This would be followed by the amino acid degradation pathway to alkanes described above (ADO or CvFAP versions), eliminating only the initial biocatalytic step catalysed by ilvE (Fig. 2). So far, this proposed pathway has not been tested In vivo, likely due to the availability of simpler and more effective routes to butane via CvFAP direct decarboxylation of valeric acid or the de novo isoleucine to α-methylbutyrate pathway (Fig. 4).

The second proposed route begins with the upregulation of L-threonine dehydratase (ilvA) to deaminate threonine to α-ketobutyrate [72]. Carbon chain elongation then follows two stages to generate α-ketocaproate, catalysed by the isopropyl malate synthase, dehydrogenase & isomerase complex (LeuABCD; [73]) (Fig. 2). The remaining steps mimic the isoleucine to butane pathway using enzymes BCKDHAB, YciA and CvFAP, to generate pentanoyl-CoA, valeric acid and butane, respectively. These latter precursors are actually structural isomers (chain isomerisation) of the equivalent compounds generated via the isoleucine degradation pathway (α-methylbutyryl-CoA and α-methylbutyric acid, respectively). Interestingly, this same LeuABCD-dependent pathway could potentially be utilised with valine to generate isobutane instead of propane, the only difference being a substitution of ilvA for ilvE (Fig. 2). This LeuABCD route from threonine to butane was investigated recently, but poor expression of a functional LeuABCD complex in *E.* *coli* prevented its implementation [5].

Pathways derived from amino acid biosynthesis and/or degradation have been shown to be viable alternatives to propane, butane and isobutane production. In particular, In vivo fermentations with supplemental amino acids yielded titres comparable to those seen with one step CvFAP-dependent fatty acid decarboxylation, without the disadvantages of VFA cytotoxicity. Therefore, commercial exploitation of amino acid based alkane gas production may be feasible, given the global abundance of food and other proteinaceous waste that could act as precursor and carbon sources.

## Conclusions and Future Perspectives

The commercialisation of production methods for biologically-sourced gaseous fuels is crucial to support the global challenges of realising renewable energy supplies and reducing the carbon footprint and other pollutants. Further research is needed to develop tuneable alkane production across the spectrum of short to very long chain hydrocarbons, effectively converting microorganisms into the ‘oilfields of the future’. This will satisfy the demand for blending with or even replacing the current dependence on petroleum-based fuels and synthetic precursors.

The transition from ‘proof-of-principle’ research to successful commercialisation requires a detailed understanding of the techno-economic factors associated with scaling biological processes. A recent study into the commercial potential of fermentative alkane gas production identified key parameters that needed optimisation to enable cost-effective fuel production, and proposed mitigations to overcome these barriers [6]. These mitigations included the transition towards robust industrial microorganisms requiring drastically reduced capital and running costs, sourcing low cost and renewable energy sources, and increasing gas production titres. The latter is particularly important for biological alkane production as the terminal ADO / CvFAP-dependent deformylation / decarboxylation step is thought to be the rate limiting step.

Identification of the important barriers to commercial success can help focus further research, for example to improve In vivo biocatalytic efficiencies, by applying enzyme evolution or redesign strategies to increase reaction rates, stability and expression within a chosen chassis. The advent of synthetic biology techniques enables more in-depth optimisation of process development beyond traditional enzyme redesign. Enhancements in productivity can be obtained by optimising DNA regulatory parts [74], both on a transcriptional and translational level [75, 76], metabolic engineering of auxiliary supply pathways to relieve bottlenecks, and the elimination or downregulation of competing side-reactions. These are important areas of process optimisation realised through bioengineering but overall process optimisation will be essential beyond the need to improve microbial cell factories for bio alkane gas production.

The unprecedented curtailment of global economic activity and mobility during early 2020 due to the Covid 19 pandemic has reduced global energy demand by 3.8% relative to the same time period in 2019 [77]. In spite of this, fossil fuel supplies remain limited and non-renewable, with demand still at high levels. The development of (ultimately) sustainable bio-manufacturing of gaseous hydrocarbons is therefore timely, with success measured by the ability to compete on price and abundancy with existing non-renewable and commercial synthesis routes.

## Data Availability

Not applicable.

## Abbreviations

*Acc*: Acetyl-CoA carboxylase
ADH: Aldehyde dehydrogenase homologue
*AdhE2*: Bi-functional aldehyde-alcohol dehydrogenases
*ADO/Ferr*: Aldehyde-deformylating oxygenase and electron transfer partner ferredoxin
*alsS*: Acetolactate synthase
*AtoB*: Acetyl-CoA acetyltransferase
*bcd*: Butyryl-CoA dehydrogenase
*BktB*: β-Ketothiolase
*BCKDHAB*: Human 2-oxoisovalerate dehydrogenase
*CAR/sfp*: Carboxylic acid reductase and activating enzyme 4′-phosphopantetheinyl transferase
*Crt*: 3-Hydroxybutyryl-CoA dehydratase
*CvFAP*: Fatty acid photodecarboxylase
*FabA*: β-Hydroxyacyl-ACP dehydrase
*FabDH*: Malonyl-CoA-ACP transacetylase and β-ketoacyl-ACP synthase
*FabG*: β-Ketoacyl-ACP reductase
*FabI*: Enoyl-ACP reductase
*Hbd*: 3-Hydroxybutyryl-CoA dehydrogenase
*ilvA*: l-Threonine dehydratase biosynthetic
*ilvC*: Ketol-acid reductoisomerase
*ilvD*: Dihydroxy-acid dehydratase
*ilvE*: Branched-chain amino acid aminotransferase
*KdcA*: Branched-chain keto acid decarboxylase
*LeuABCD*: Isopropyl malate synthase, dehydrogenase & isomerases complex
*NphT7*: Acetoacetyl CoA synthase
*phaB*: Acetoacetyl-CoA reductase
*phaJ4b*: R-specific enoyl CoA hydratase 4b
*PyDH*: Pyruvate dehydrogenase
*Ter*: Trans-2-enoyl-CoA reductase
*Tes4*: Acyl-ACP thioesterase
*thl*: Thiolase
*YciA*: Acyl-CoA thioester hydrolase
